# {1-[1-(2-Hy­droxy­phen­yl)ethyl­idene]-2-(pyridin-2-yl-κ*N*)hydrazine-κ^2^ *N*′,*O*}{1-[1-(2-oxidophen­yl)ethyl­idene]-2-(pyridin-2-yl-κ*N*)hydrazine-κ^2^ *N*′,*O*}nickelate(II) nitrate hemihydrate

**DOI:** 10.1107/S2056989018005261

**Published:** 2018-04-06

**Authors:** Sarr Mamour, Diop Mayoro, Thiam Elhadj Ibrahima, Gaye Mohamed, Barry Aliou Hamady, Javier Ellena

**Affiliations:** a Département de Chimie, Faculté des Sciences et Techniques, Université Cheikh Anta Diop, Dakar, Senegal; b Département de Chimie, Faculté des Sciences, Université de Nouakchott, Nouakchott, Mauritanie; c Instituto de Física de São Carlos, Universidade de São Paulo, CP 369, São Carlos, SP, Brazil

**Keywords:** crystal structure, nickel, Schiff base

## Abstract

The 2-hydrazino­pyridine precursor has been widely used to prepare ligands of various kinds by condensation with carbonyl compounds. These types of ligands are suitable for synthesizing novel transition metal (II) complexes with inter­esting magnetic properties. In this context we have synthesized the ligand 1-(2-hy­droxy­phenyl-2-ethyl­idene)-2-(pyridin-2-yl)hydrazine (H*L*) which was used in the preparation of the mononuclear title complex, [Ni(C_13_H_12_N_3_O)(C_13_H_13_N_3_O)]NO_3_·0.5H_2_O. As a result of the presence of H*L* and *L* in the [{Ni(H*L*)(*L*)}]^+^ unit, the complex appears to be a supramolecular dimer composed of the Δ(−) and Λ(−) optical isomers, which are linked by strong hydrogen-bonds. As well as the dimer generated by two mononuclear [{Ni(H*L*)(*L*)}]^+^ cations, the asymmetric unit also contains two nitrate anions and one water mol­ecule. Each Ni atom is coordinated to two ligand mol­ecules by a nitro­gen atom of the pyridine ring, an imine nitro­gen atom and a phenolic oxygen atom of one of the ligand mol­ecules and a phenolate oxygen atom of the other organic mol­ecules. The environment around the cation is a distorted octa­hedron. The basal planes are defined by the two nitro­gen atoms of the pyridine rings and the two phenolic oxygen atoms of the ligand, the apical positions being occupied by the azomethine atoms. The O atoms of one of the nitrate ions are disordered over two sets of sites in a 0.745 (9):0.255 (9) ratio. In the crystal, the dimers are linked by numerous hydrogen bonds, forming a three-dimensional framework.

## Abstract 2

The asymmetric unit of the title complex comprises a supramolecular dimer composed of the Δ(−) and Λ(−) optical isomers, which are linked by strong hydrogen-bonds, two nitrate anions and one water mol­ecule. In the crystal, the dimers are joined by numerous hydrogen bonds, forming a three-dimensional framework.

## Chemical context   

Organic ligands derived from salicyl­aldehyde containing N and O donor atoms are widely used in coordination chemistry (Wang *et al.*, 2006 ▸; Güveli & Ülküseven, 2011 ▸; Liu *et al.*, 2018 ▸). Indeed, these derivatives can give very different structures depending on the type of metal used and the reaction medium (Mahapatra *et al.*, 2016 ▸). The coordination chemistry of trans­ition metals continues to be widely explored by researchers because of the wide variety of structures (Bhatta­charya & Mohanta, 2015 ▸) and applications of these derivatives in different fields (El-Sayed *et al.*, 2016 ▸; Donga *et al.*, 2016 ▸). The growing inter­est in the use in coordination chemistry of ligands containing a hydrazino unit (Drożdżewski & Kubiak, 2009 ▸; Mukherjee *et al.*, 2013 ▸; Guhathakurta *et al.*, 2017 ▸) is due to the presence of N donor atoms, allowing them to act as multidentate ligands to generate supra­molecular structures (Konar, 2015 ▸; Chavan *et al.*, 2014 ▸) that have inter­esting catalytic properties (Nassar *et al.*, 2017 ▸) or biological activities (Singh *et al.*, 2013 ▸). In this context we have synthesized the ligand 1-(2-hy­droxy­phenyl-2-ethyl­idene)-2-(pyridin-2-yl)hydrazine (H*L*), which was used in the preparation of the title compound. We combined 2-hy­droxy­aceto­phenone and 2-hydrazino pyridine to prepare a ligand with four potential donor sites (N, O) that acts as a tridentate ligand. In trying to coordinate the 1-(2-hy­droxy­phenyl-2-ethyl­idene)-2-(pyridin-2-yl)hydrazine ligand to the first series of transition metals in ethanol, we obtained a nickel(II) complex.
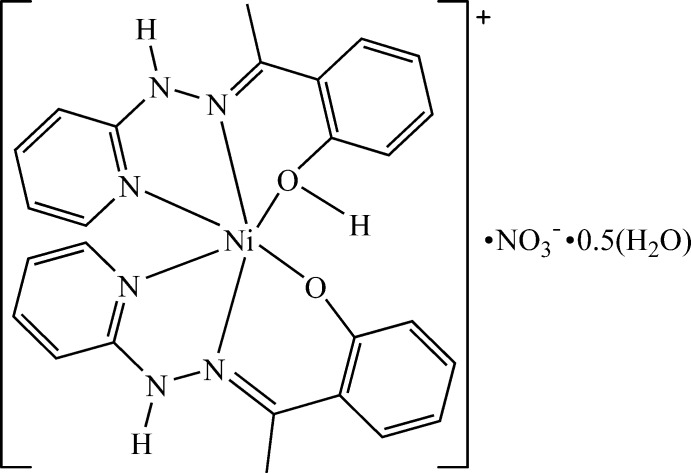


## Structural commentary   

**Figure 1:**
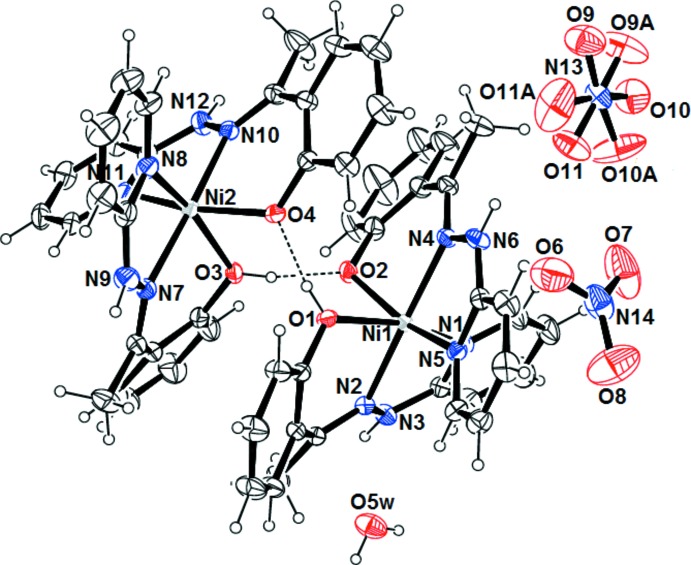
An *ORTEP* view of the title compound, showing the atom-numbering scheme and intra­molecular contacts (Table 2 ▸) as dashed lines. Displacement ellipsoids are plotted at the 50% probability level.

Fig. 1 ▸ shows the structure of the complex. The asymmetric unit contains a dimer generated by two mononuclear [{Ni(H*L*)(*L*)}]^+^ cations, which are strongly hydrogen bonded, two nitrate anions and one water mol­ecule. The O atoms of one of the nitrate ions are disordered over two sets of sites in a 0.745 (9):0.255 (9) ratio. As a result of the presence of H*L* and *L* in the [{Ni(H*L*)(*L*)}]^+^ unit, the complex is chiral. The dimer is formed by the Δ(−) and Λ(−) optical isomers because of the clockwise and anti-clockwise arrangement of the ligands around the Ni^2+^ ion. The two optical isomers of the dimer are linked by strong O—H⋯O hydrogen bonds between the phenoxo oxygen atoms and the phenolic hydrogen atoms (O1—H1*O*⋯O4 and O3—H3*O*⋯O2) with a mean H⋯*A* distances of 1.64 Å.

In both complex mol­ecules, the Ni^2+^ ion is hexa­coordinated in an octa­hedral environment. Each Ni^2+^ ion is bonded to a ligand mol­ecule, whose phenolic function is deprotonated and to a second neutral ligand mol­ecule. The basal plane of the octa­hedron around each Ni^2+^ ion is occupied by two nitro­gen atoms from the pyridine moieties, a phenolic oxygen atom and a phenolate oxygen atom. The apical positions are occupied by the nitro­gen atoms of the imine functions. The angles (Table 1 ▸) in the basal plane of the octa­hedron are in the range 84.34 (6)–102.46 (7)° for Ni1 and 84.32 (6)–103.78 (7)° for Ni2. The sum of the angles around Ni1 and Ni2 are respectively 363.44° and 363.90° indicating deformation of the octa­hedron. The angles formed by the axial atoms around Ni1 and Ni2 (N2—Ni1—N4 and N7—Ni2—N10) deviate from the ideal value of 180°. The Ni—O/N bond lengths are similar to the observed distances in hexa­dentate nickel(II) complex [Ni(*L*)_2_] where H*L* is 2-[(piperidin-2-yl­methyl­imino)­meth­yl]phenol (Jana *et al.*, 2017 ▸). The diagonal basal angles (N1—Ni1—O1, N5—Ni1—O2, N8—Ni2—O3 and N11—Ni2—O4) and the apical angles (N2—Ni1—N4 and N7—Ni2—N10) deviate significantly from the ideal values of 180°. The angles N2—Ni1—O1 and N2—Ni1—N1 are very different. This can be explained by the rings formed by the ligand by binding in a tridentate fashion to the Ni^2+^ ion. The first angle is derived from a six-membered ring whereas the second one is derived from a five-membered ring. The flexibility of the six-membered ring compared to the five-membered ring implies that the angles should be larger in the six-membered ring than in the five-membered ring. The same behavior is observed for the angles around Ni1 with the second ligand mol­ecule. These observations are also noticed for the second mol­ecule in the asymmetric unit.

## Supra­molecular features   

**Figure 2:**
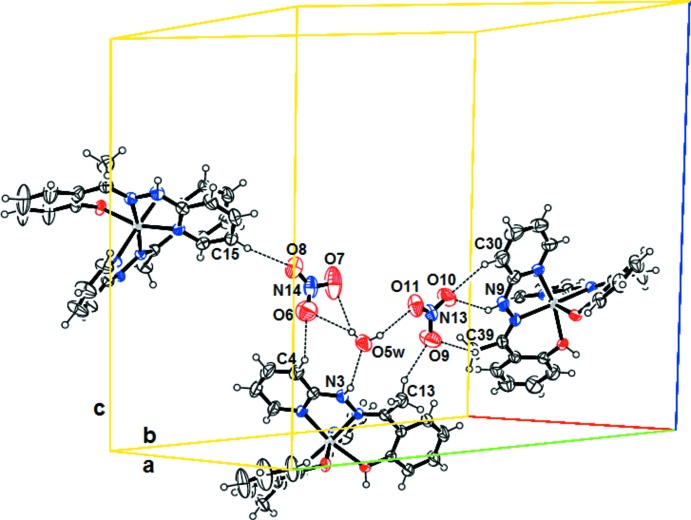
Mol­ecular representation of the title compound, showing the inter­molecular hydrogen-bond contacts (Table 2 ▸) as dotted lines.

In the crystal, the complex appears as a dimer composed by the Δ(−) and Λ(−) optical isomers, which are linked by strong hydrogen bonds (Table 2 ▸). The dimers are linked by different inter­molecular hydrogen bonds, O*W*—H⋯ONO_2_, N—H⋯ONO_2_, N—H⋯O*W* and C—H⋯ONO_2_, involving the complex mol­ecule, the non-coordinating water mol­ecule and the uncoordinated nitrate groups (Fig. 2 ▸). These inter­molecular and intra­molecular hydrogen bonds stabilize and link the components into a three-dimensional network.

## Synthesis and crystallization   

A mixture of 2-hydrazino­pyridine (1 mmol) and 2-hydroxy­aceto­phenone (1 mmol) in ethanol (10 mL) was stirred under reflux for 60 min. On cooling, a yellow precipitate was obtained. After filtration, the resulting solid was dried in a desiccator. C_13_H_13_N_3_O (H*L*), yield 60%, m.p. 388 K. Calculated: C, 68.70; H, 5.77; N, 18.49. Found: C, 68.72; H, 5.76; N, 18.46%. IR (cm^−1^): 3289 (ν O—H), 3051 (ν N—H), 1514 (ν C=N), 1576, 1507, 1493, 1247 (ν C—O), 1145, 1043 (ν N—N), 756. ^1^H NMR: δ (ppm): 2.3 (3H, *s*, —CH_3_), 6.79–6.85 (8H, H—Ph and H—Py), 8.7 (1H, *s*, H—N), 12.9 (1H, *br*, H—O). ^13^C NMR: δ(ppm): 12, 107, 116, 117, 118, 119, 120, 127, 130, 138, 149, 156, 158. A mixture of NiCl_2_·6H_2_O (1 mmol) in ethanol (10 mL) was added to a solution of H*L* (2 mol) in 10 mL of ethanol. The mixture was stirred for 60 min and the resulting greenish solution was filtered. The filtrate was kept at 298 K and after six days, green crystals suitable for X-ray analysis appeared and were collected by filtration. [C_26_H_26_N_7_NiO_5.5_], yield 40%. Calculated: C, 53.54; H, 4.49; N, 16.81. Found: C, 53.50; H, 4.52; N, 16.76%. μ_eff_ (mB): 1.8. Λ_M_ (S cm^2^ mol^−1^): 5. IR (cm^−1^): 3289, 3051, 3289, 1614, 1576, 1375, 1229, 1015.

## Refinement   

Crystal data, data collection and structure refinement details are summarized in Table 3 ▸. H atoms of OH and OH_2_ groups were located in difference-Fourier maps and refined using a riding model with *U*_iso_(H) = 1.5*U*_eq_(O). Other H atoms (CH, NH and CH_3_ groups) were geometrically optimized (C—H = 0.93–0.96 Å, Å N—H = 0.86 Å) and refined as riding with *U*_iso_(H) = 1.5*U*_eq_(C-meth­yl) and 1.2*U*_eq_(C) for all other H atoms. High thermal motion for the O atoms of one of the nitrate group was noted, indicating some disorder in their positions. Each of these O atoms was distributed over two sites with a refined occupancy ratio of 0.745 (9):0.255 (9).

## Supplementary Material

Crystal structure: contains datablock(s) I. DOI: 10.1107/S2056989018005261/tx2004sup1.cif

CCDC reference: 1834549

Additional supporting information:  crystallographic information; 3D view; checkCIF report

## Figures and Tables

**Table 1:** Selected geometric parameters (Å, °)

Ni1—O2	2.0371 (14)	Ni2—O4	2.0336 (14)
Ni1—N4	2.0388 (16)	Ni2—N10	2.0337 (17)
Ni1—O1	2.0483 (13)	Ni2—N7	2.0455 (17)
Ni1—N1	2.0500 (16)	Ni2—N8	2.0594 (18)
Ni1—N2	2.0501 (16)	Ni2—N11	2.0606 (17)
Ni1—N5	2.0564 (17)	Ni2—O3	2.0667 (14)
			
O1—Ni1—N1	165.40 (6)	O2—Ni1—N5	160.88 (6)
N4—Ni1—N2	173.93 (6)	N10—Ni2—N7	176.60 (7)
O1—Ni1—N2	86.53 (6)	O4—Ni2—N11	163.23 (6)
N1—Ni1—N2	79.29 (6)	N8—Ni2—O3	160.88 (7)

**Table 2:** Hydrogen-bond geometry (Å, °)

*D*—H⋯*A*	*D*—H	H⋯*A*	*D*⋯*A*	*D*—H⋯*A*
O1—H1*O*⋯O4	0.82	1.62	2.4093 (18)	161
O3—H3*O*⋯O2	0.82	1.66	2.4647 (19)	167
O5*W*—H5*WA*⋯O11^i^	0.84	2.16	2.979 (5)	165
O5*W*—H5*WA*⋯O11*A* ^i^	0.84	1.94	2.767 (14)	170
O5*W*—H5*WB*⋯O6^i^	0.74	2.47	3.142 (4)	153
O5*W*—H5*WB*⋯O7^i^	0.74	2.42	3.094 (5)	153
N3—H3*N*⋯O5*W*	0.86	2.23	2.933 (2)	139
N6—H6*N*⋯O11	0.86	2.19	2.991 (4)	156
N6—H6*N*⋯O11*A*	0.86	2.62	3.47 (3)	172
N9—H9*N*⋯O10^ii^	0.86	2.30	3.041 (4)	145
N9—H9*N*⋯O9*A* ^ii^	0.86	2.26	3.107 (12)	167
N12—H12*N*⋯O6^iii^	0.86	2.13	2.961 (3)	162
C2—H2⋯O10^iv^	0.93	2.63	3.437 (5)	146
C4—H4⋯O6^i^	0.93	2.56	3.409 (4)	152
C13—H13*C*⋯O9^i^	0.96	2.33	3.231 (5)	156
C15—H15⋯O8^v^	0.93	2.62	3.544 (4)	170
C26—H26*C*⋯O10*A*	0.96	2.59	3.332 (18)	134
C28—H28⋯O10*A* ^vi^	0.93	2.64	3.104 (12)	111
C30—H30⋯O10^ii^	0.93	2.33	3.117 (5)	142
C39—H39*A*⋯O9*A* ^ii^	0.96	2.39	2.938 (11)	116

Symmetry codes: (i) 
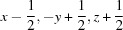
; (ii) 
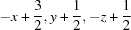
; (iii) 
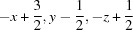
; (iv) 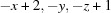; (v) 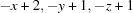; (vi) 
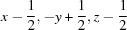
.

**Table 3:** Experimental details

Crystal data	
Chemical formula	[Ni(C_13_H_12_N_3_O)(C_13_H_13_N_3_O)]NO_3_·0.5H_2_O
*M*_r_	583.25
Crystal system, space group	Monoclinic, *P*2_1_/*n*
Temperature (K)	293
*a*, *b*, *c* (Å)	16.1988 (3), 18.5375 (3), 17.9175 (3)
β (°)	97.5822 (18)
*V* (Å^3^)	5333.30 (17)
*Z*	8
Radiation type	Mo *K*α
μ (mm^−1^)	0.78
Crystal size (mm)	0.08 × 0.07 × 0.06

Data collection	
Diffractometer	Nonius KappaCCD
No. of measured, independent and observed [*I* > 2σ(*I*)] reflections	150855, 13035, 10488
*R*_int_	0.048
(sin θ/λ)_max_ (Å^−1^)	0.683

Refinement	
*R*[*F* ^2^ > 2σ(*F* ^2^)], *wR*(*F* ^2^), *S*	0.040, 0.107, 1.05
No. of reflections	13035
No. of parameters	745
H-atom treatment	H atoms treated by a mixture of independent and constrained refinement
Δρ_max_, Δρ_min_ (e Å^−3^)	0.44, −0.35

Computer programs: *APEX3* and *SAINT* (Bruker, 2016 ▸), *SHELXT* (Sheldrick, 2015*a* ▸), *SHELXL2014* (Sheldrick, 2015*b* ▸) and *ORTEP-3 for Windows* (Farrugia, 2012 ▸).

## supplementary crystallographic information

### Crystal data

**Table:**

[Ni(C_13_H_12_N_3_O)(C_13_H_13_N_3_O)]NO_3_·0.5H_2_O	*F*(000) = 2424
*M**_r_* = 583.25	*D*_x_ = 1.453 Mg m^−^^3^
Monoclinic, *P*2_1_/*n*	Mo *K*α radiation, λ = 0.71073 Å
*a* = 16.1988 (3) Å	Cell parameters from 4920 reflections
*b* = 18.5375 (3) Å	θ = 2.4–28.6°
*c* = 17.9175 (3) Å	µ = 0.78 mm^−^^1^
β = 97.5822 (18)°	*T* = 293 K
*V* = 5333.30 (17) Å^3^	Prismatic, green
*Z* = 8	0.08 × 0.07 × 0.06 mm

### Data collection

**Table:**

Nonius KappaCCD diffractometer	10488 reflections with *I* > 2σ(*I*)
Radiation source: fine-focus sealed tube	*R*_int_ = 0.048
Detector resolution: 9 pixels mm^-1^	θ_max_ = 29.0°, θ_min_ = 3.4°
CCD scans	*h* = −21→21
150855 measured reflections	*k* = −25→22
13035 independent reflections	*l* = −24→24

### Refinement

**Table:**

Refinement on *F*^2^	Primary atom site location: structure-invariant direct methods
Least-squares matrix: full	Secondary atom site location: difference Fourier map
*R*[*F*^2^ > 2σ(*F*^2^)] = 0.040	Hydrogen site location: inferred from neighbouring sites
*wR*(*F*^2^) = 0.107	H atoms treated by a mixture of independent and constrained refinement
*S* = 1.05	*w* = 1/[σ^2^(*F*_o_^2^) + (0.046*P*)^2^ + 3.4265*P*] where *P* = (*F*_o_^2^ + 2*F*_c_^2^)/3
13035 reflections	(Δ/σ)_max_ = 0.002
745 parameters	Δρ_max_ = 0.44 e Å^−^^3^
0 restraints	Δρ_min_ = −0.35 e Å^−^^3^

### Special details

**Table:**

Geometry. All esds (except the esd in the dihedral angle between two l.s. planes) are estimated using the full covariance matrix. The cell esds are taken into account individually in the estimation of esds in distances, angles and torsion angles; correlations between esds in cell parameters are only used when they are defined by crystal symmetry. An approximate (isotropic) treatment of cell esds is used for estimating esds involving l.s. planes.

### Fractional atomic coordinates and isotropic or equivalent isotropic displacement parameters (Å^2^)

**Table:**

	*x*	*y*	*z*	*U*_iso_*/*U*_eq_	Occ. (<1)
Ni1	0.69969 (2)	0.20122 (2)	0.45200 (2)	0.02911 (7)	
Ni2	0.48759 (2)	0.23628 (2)	0.21983 (2)	0.03373 (7)	
O1	0.65123 (9)	0.28020 (7)	0.37910 (7)	0.0355 (3)	
H1O	0.639283	0.277592	0.333265	0.053*	
O2	0.60421 (8)	0.13968 (7)	0.40125 (8)	0.0387 (3)	
O3	0.48693 (9)	0.20577 (8)	0.33069 (8)	0.0398 (3)	
H3O	0.521775	0.184018	0.359604	0.060*	
O4	0.61270 (9)	0.24528 (8)	0.24967 (8)	0.0380 (3)	
O5W	0.53344 (14)	0.20867 (11)	0.71451 (12)	0.0733 (6)	
H5WA	0.519783	0.248203	0.731618	0.110*	
H5WB	0.553843	0.184750	0.744202	0.110*	
O6	1.0763 (2)	0.43370 (16)	0.30666 (18)	0.1090 (9)	
O7	1.10911 (18)	0.34282 (14)	0.3732 (3)	0.1389 (14)	
O8	1.1199 (3)	0.44896 (18)	0.41916 (18)	0.1352 (13)	
O9	1.0251 (3)	0.1008 (3)	0.2123 (2)	0.1010 (16)	0.745 (9)
O10	1.0324 (3)	0.05074 (18)	0.3176 (2)	0.0912 (16)	0.745 (9)
O11	0.9948 (3)	0.1624 (2)	0.3030 (3)	0.0828 (16)	0.745 (9)
N1	0.72332 (10)	0.12931 (9)	0.53913 (9)	0.0371 (4)	
N2	0.62439 (10)	0.24142 (8)	0.52552 (9)	0.0317 (3)	
N3	0.60892 (11)	0.18806 (9)	0.57598 (10)	0.0387 (4)	
H3N	0.565592	0.188957	0.599013	0.046*	
N4	0.77212 (10)	0.15067 (9)	0.38318 (9)	0.0350 (3)	
N5	0.80350 (10)	0.26613 (9)	0.46670 (9)	0.0349 (3)	
N6	0.84061 (11)	0.19129 (10)	0.37336 (11)	0.0440 (4)	
H6N	0.873821	0.178285	0.342155	0.053*	
N7	0.45535 (11)	0.33573 (9)	0.25612 (10)	0.0402 (4)	
N8	0.50166 (12)	0.29814 (10)	0.12688 (10)	0.0430 (4)	
N9	0.47946 (13)	0.38903 (10)	0.21018 (12)	0.0517 (5)	
H9N	0.480876	0.433502	0.223946	0.062*	
N10	0.51236 (11)	0.13578 (9)	0.18304 (9)	0.0365 (4)	
N11	0.36844 (11)	0.19595 (9)	0.19645 (10)	0.0389 (4)	
N12	0.44383 (11)	0.09206 (10)	0.18355 (11)	0.0452 (4)	
H12N	0.448115	0.045896	0.181277	0.054*	
N13	1.01909 (13)	0.10474 (11)	0.28065 (13)	0.0515 (5)	
N14	1.10515 (16)	0.40819 (13)	0.3660 (2)	0.0734 (7)	
C1	0.78089 (15)	0.07688 (14)	0.54885 (14)	0.0531 (6)	
H1	0.820972	0.074866	0.516225	0.064*	
C2	0.7832 (2)	0.02635 (17)	0.60461 (17)	0.0732 (9)	
H2	0.823550	−0.009617	0.609891	0.088*	
C3	0.7236 (2)	0.03046 (17)	0.65275 (17)	0.0781 (9)	
H3	0.723264	−0.003683	0.690729	0.094*	
C4	0.66526 (19)	0.08390 (14)	0.64546 (15)	0.0604 (7)	
H4	0.625565	0.087153	0.678380	0.072*	
C5	0.66688 (13)	0.13354 (11)	0.58715 (11)	0.0387 (4)	
C6	0.57756 (12)	0.29756 (11)	0.51850 (11)	0.0349 (4)	
C7	0.59975 (12)	0.35707 (10)	0.46981 (11)	0.0350 (4)	
C8	0.58773 (15)	0.42805 (12)	0.49327 (13)	0.0476 (5)	
H8	0.565982	0.435732	0.538118	0.057*	
C9	0.60724 (19)	0.48666 (13)	0.45176 (16)	0.0599 (7)	
H9	0.600129	0.533224	0.469160	0.072*	
C10	0.63726 (19)	0.47584 (13)	0.38453 (15)	0.0582 (6)	
H10	0.648914	0.515246	0.355545	0.070*	
C11	0.65030 (15)	0.40670 (11)	0.35960 (13)	0.0456 (5)	
H11	0.670248	0.400100	0.313735	0.055*	
C12	0.63392 (12)	0.34669 (10)	0.40234 (11)	0.0331 (4)	
C13	0.50247 (15)	0.30578 (13)	0.55871 (14)	0.0508 (6)	
H13A	0.474444	0.260221	0.559582	0.076*	
H13B	0.465320	0.340702	0.532896	0.076*	
H13C	0.519620	0.321690	0.609375	0.076*	
C14	0.81372 (14)	0.32814 (12)	0.50585 (12)	0.0415 (5)	
H14	0.778149	0.337685	0.541360	0.050*	
C15	0.87401 (15)	0.37803 (13)	0.49606 (14)	0.0498 (5)	
H15	0.878855	0.420641	0.523682	0.060*	
C16	0.92728 (16)	0.36294 (13)	0.44385 (15)	0.0554 (6)	
H16	0.969511	0.395254	0.436867	0.066*	
C17	0.91832 (15)	0.30082 (13)	0.40239 (14)	0.0508 (6)	
H17	0.953668	0.290484	0.366905	0.061*	
C18	0.85450 (12)	0.25329 (11)	0.41485 (12)	0.0369 (4)	
C19	0.75990 (14)	0.08936 (11)	0.34878 (12)	0.0422 (5)	
C20	0.69007 (15)	0.04357 (11)	0.36439 (13)	0.0467 (5)	
C21	0.61659 (14)	0.06938 (11)	0.38945 (14)	0.0464 (5)	
C22	0.55573 (19)	0.02025 (15)	0.4040 (2)	0.0843 (11)	
H22	0.506963	0.037178	0.420060	0.101*	
C23	0.5661 (2)	−0.05275 (17)	0.3953 (3)	0.1195 (18)	
H23	0.524571	−0.084533	0.405414	0.143*	
C24	0.6370 (3)	−0.07860 (16)	0.3718 (3)	0.1123 (16)	
H24	0.644130	−0.128024	0.366466	0.135*	
C25	0.6982 (2)	−0.03155 (14)	0.3560 (2)	0.0779 (9)	
H25	0.746026	−0.049826	0.339357	0.093*	
C26	0.8182 (2)	0.06368 (15)	0.29570 (17)	0.0663 (8)	
H26A	0.829879	0.102640	0.263369	0.099*	
H26B	0.792852	0.024667	0.265758	0.099*	
H26C	0.869219	0.047331	0.324078	0.099*	
C27	0.52472 (17)	0.27665 (14)	0.06079 (13)	0.0534 (6)	
H27	0.525700	0.227499	0.050559	0.064*	
C28	0.5468 (2)	0.32390 (17)	0.00792 (16)	0.0685 (8)	
H28	0.562676	0.307340	−0.037071	0.082*	
C29	0.5447 (2)	0.39698 (18)	0.02355 (18)	0.0765 (9)	
H29	0.558819	0.430264	−0.011499	0.092*	
C30	0.5221 (2)	0.42044 (15)	0.09038 (17)	0.0667 (7)	
H30	0.520610	0.469412	0.101392	0.080*	
C31	0.50121 (15)	0.36893 (12)	0.14158 (13)	0.0470 (5)	
C32	0.41969 (14)	0.35265 (12)	0.31432 (13)	0.0466 (5)	
C33	0.38847 (14)	0.29495 (13)	0.35971 (12)	0.0461 (5)	
C34	0.42222 (13)	0.22454 (13)	0.36726 (12)	0.0421 (5)	
C35	0.38711 (17)	0.17403 (17)	0.41051 (16)	0.0631 (7)	
H35	0.410490	0.128223	0.416399	0.076*	
C36	0.3180 (2)	0.1903 (2)	0.44514 (19)	0.0794 (9)	
H36	0.295403	0.155669	0.474095	0.095*	
C37	0.28280 (19)	0.2574 (2)	0.43673 (19)	0.0772 (9)	
H37	0.235496	0.268162	0.458891	0.093*	
C38	0.31768 (17)	0.30871 (17)	0.39546 (16)	0.0627 (7)	
H38	0.293673	0.354339	0.390905	0.075*	
C39	0.4058 (2)	0.43043 (15)	0.33345 (18)	0.0737 (8)	
H39A	0.457030	0.456636	0.334309	0.111*	
H39B	0.387095	0.433267	0.382026	0.111*	
H39C	0.364409	0.450980	0.296217	0.111*	
C40	0.29525 (14)	0.22856 (13)	0.20163 (13)	0.0459 (5)	
H40	0.294483	0.278430	0.207136	0.055*	
C41	0.22184 (15)	0.19154 (15)	0.19913 (14)	0.0543 (6)	
H41	0.172469	0.215533	0.204218	0.065*	
C42	0.22279 (16)	0.11739 (15)	0.18885 (15)	0.0572 (6)	
H42	0.173632	0.091090	0.186558	0.069*	
C43	0.29586 (15)	0.08315 (13)	0.18210 (14)	0.0490 (5)	
H43	0.297188	0.033645	0.173922	0.059*	
C44	0.36880 (13)	0.12425 (11)	0.18777 (11)	0.0396 (4)	
C45	0.58119 (14)	0.10846 (12)	0.16676 (13)	0.0449 (5)	
C46	0.65453 (14)	0.15492 (12)	0.16520 (12)	0.0413 (5)	
C47	0.66935 (13)	0.21875 (11)	0.20858 (11)	0.0362 (4)	
C48	0.74569 (15)	0.25429 (13)	0.20936 (14)	0.0486 (5)	
H48	0.756683	0.294947	0.239437	0.058*	
C49	0.80495 (17)	0.23040 (14)	0.16657 (17)	0.0607 (7)	
H49	0.855829	0.254120	0.169040	0.073*	
C50	0.78884 (18)	0.17125 (15)	0.12000 (17)	0.0648 (7)	
H50	0.827562	0.156359	0.089227	0.078*	
C51	0.71492 (17)	0.13459 (14)	0.11962 (15)	0.0567 (6)	
H51	0.704525	0.094891	0.088037	0.068*	
C52	0.58736 (19)	0.02966 (15)	0.1491 (2)	0.0841 (11)	
H52A	0.569116	0.001707	0.189008	0.126*	
H52B	0.644130	0.017748	0.144472	0.126*	
H52C	0.552796	0.019100	0.102696	0.126*	
O9A	1.0433 (9)	0.0516 (6)	0.2558 (10)	0.123 (7)	0.255 (9)
O10A	1.0180 (12)	0.1012 (10)	0.3517 (6)	0.150 (8)	0.255 (9)
O11A	0.9874 (13)	0.1541 (8)	0.2537 (12)	0.153 (9)	0.255 (9)

### Atomic displacement parameters (Å^2^)

**Table:**

	*U*^11^	*U*^22^	*U*^33^	*U*^12^	*U*^13^	*U*^23^
Ni1	0.02986 (12)	0.02894 (12)	0.02853 (12)	0.00262 (9)	0.00381 (9)	−0.00052 (9)
Ni2	0.03638 (14)	0.03074 (13)	0.03250 (13)	0.00373 (10)	−0.00127 (10)	−0.00269 (9)
O1	0.0496 (8)	0.0289 (6)	0.0266 (6)	0.0042 (6)	0.0001 (6)	−0.0027 (5)
O2	0.0348 (7)	0.0267 (7)	0.0518 (8)	0.0030 (5)	−0.0046 (6)	−0.0034 (6)
O3	0.0345 (7)	0.0490 (8)	0.0353 (7)	0.0127 (6)	0.0028 (6)	0.0046 (6)
O4	0.0363 (7)	0.0461 (8)	0.0315 (7)	0.0012 (6)	0.0040 (6)	−0.0088 (6)
O5W	0.0981 (16)	0.0613 (12)	0.0682 (12)	0.0073 (11)	0.0399 (12)	0.0035 (10)
O6	0.140 (3)	0.0916 (19)	0.097 (2)	0.0133 (18)	0.0251 (18)	−0.0098 (16)
O7	0.0893 (19)	0.0549 (15)	0.268 (4)	0.0096 (13)	0.005 (2)	0.013 (2)
O8	0.195 (4)	0.099 (2)	0.105 (2)	−0.058 (2)	−0.003 (2)	−0.0120 (18)
O9	0.142 (3)	0.093 (4)	0.075 (2)	0.015 (2)	0.043 (2)	0.013 (2)
O10	0.133 (3)	0.056 (2)	0.084 (3)	0.0132 (19)	0.011 (2)	0.026 (2)
O11	0.091 (3)	0.0467 (18)	0.121 (4)	0.0005 (17)	0.053 (3)	−0.014 (2)
N1	0.0361 (9)	0.0389 (9)	0.0359 (9)	0.0049 (7)	0.0034 (7)	0.0056 (7)
N2	0.0337 (8)	0.0307 (8)	0.0312 (8)	−0.0021 (6)	0.0060 (6)	0.0002 (6)
N3	0.0411 (9)	0.0369 (9)	0.0412 (9)	0.0012 (7)	0.0166 (7)	0.0050 (7)
N4	0.0374 (9)	0.0336 (8)	0.0346 (8)	0.0054 (7)	0.0073 (7)	0.0003 (7)
N5	0.0341 (8)	0.0387 (9)	0.0318 (8)	−0.0005 (7)	0.0035 (6)	−0.0002 (7)
N6	0.0420 (10)	0.0464 (10)	0.0475 (10)	0.0018 (8)	0.0204 (8)	−0.0047 (8)
N7	0.0415 (9)	0.0339 (9)	0.0427 (10)	0.0052 (7)	−0.0035 (7)	−0.0043 (7)
N8	0.0510 (11)	0.0378 (9)	0.0375 (9)	−0.0021 (8)	−0.0042 (8)	0.0013 (7)
N9	0.0672 (13)	0.0289 (9)	0.0575 (12)	0.0024 (8)	0.0024 (10)	−0.0026 (8)
N10	0.0397 (9)	0.0326 (8)	0.0363 (9)	0.0009 (7)	0.0014 (7)	−0.0029 (7)
N11	0.0371 (9)	0.0414 (9)	0.0360 (9)	0.0014 (7)	−0.0039 (7)	0.0005 (7)
N12	0.0422 (10)	0.0307 (9)	0.0617 (12)	−0.0012 (7)	0.0032 (8)	−0.0024 (8)
N13	0.0512 (11)	0.0402 (11)	0.0665 (14)	0.0023 (9)	0.0201 (10)	0.0066 (10)
N14	0.0539 (13)	0.0441 (13)	0.122 (2)	0.0000 (10)	0.0122 (15)	−0.0042 (15)
C1	0.0491 (13)	0.0597 (15)	0.0508 (13)	0.0217 (11)	0.0078 (10)	0.0146 (11)
C2	0.082 (2)	0.0703 (19)	0.0685 (18)	0.0384 (16)	0.0137 (15)	0.0289 (15)
C3	0.100 (2)	0.0726 (19)	0.0644 (18)	0.0271 (18)	0.0197 (17)	0.0364 (15)
C4	0.0750 (18)	0.0571 (15)	0.0530 (14)	0.0103 (13)	0.0231 (13)	0.0195 (12)
C5	0.0429 (11)	0.0368 (10)	0.0367 (10)	0.0005 (8)	0.0062 (8)	0.0043 (8)
C6	0.0345 (10)	0.0363 (10)	0.0339 (10)	0.0020 (8)	0.0050 (8)	−0.0046 (8)
C7	0.0372 (10)	0.0307 (9)	0.0364 (10)	0.0043 (8)	0.0024 (8)	−0.0019 (8)
C8	0.0592 (14)	0.0378 (11)	0.0474 (12)	0.0085 (10)	0.0132 (10)	−0.0058 (9)
C9	0.0853 (19)	0.0295 (11)	0.0672 (16)	0.0075 (11)	0.0187 (14)	−0.0049 (11)
C10	0.0839 (19)	0.0314 (11)	0.0619 (16)	0.0009 (11)	0.0190 (13)	0.0067 (10)
C11	0.0622 (14)	0.0354 (11)	0.0408 (11)	0.0020 (10)	0.0131 (10)	0.0027 (9)
C12	0.0369 (10)	0.0288 (9)	0.0323 (9)	0.0028 (7)	0.0001 (7)	−0.0015 (7)
C13	0.0478 (13)	0.0531 (14)	0.0550 (14)	0.0140 (11)	0.0203 (11)	0.0045 (11)
C14	0.0436 (11)	0.0448 (12)	0.0347 (10)	−0.0025 (9)	0.0004 (8)	−0.0025 (9)
C15	0.0560 (14)	0.0410 (12)	0.0505 (13)	−0.0078 (10)	−0.0004 (11)	−0.0004 (10)
C16	0.0533 (14)	0.0441 (13)	0.0693 (16)	−0.0102 (11)	0.0099 (12)	0.0120 (12)
C17	0.0487 (13)	0.0508 (13)	0.0564 (14)	−0.0010 (10)	0.0200 (11)	0.0093 (11)
C18	0.0338 (10)	0.0393 (10)	0.0381 (10)	0.0037 (8)	0.0063 (8)	0.0052 (8)
C19	0.0489 (12)	0.0361 (11)	0.0416 (11)	0.0119 (9)	0.0059 (9)	−0.0026 (9)
C20	0.0543 (13)	0.0292 (10)	0.0552 (13)	0.0079 (9)	0.0018 (10)	−0.0064 (9)
C21	0.0454 (12)	0.0288 (10)	0.0621 (14)	0.0009 (9)	−0.0039 (10)	−0.0024 (9)
C22	0.0514 (15)	0.0380 (14)	0.165 (4)	−0.0042 (12)	0.0191 (19)	−0.0068 (17)
C23	0.080 (2)	0.0387 (16)	0.243 (6)	−0.0197 (16)	0.034 (3)	−0.014 (2)
C24	0.098 (3)	0.0309 (14)	0.211 (5)	−0.0014 (16)	0.033 (3)	−0.021 (2)
C25	0.077 (2)	0.0357 (13)	0.123 (3)	0.0083 (13)	0.0208 (19)	−0.0169 (15)
C26	0.0819 (19)	0.0514 (15)	0.0716 (18)	0.0102 (14)	0.0326 (15)	−0.0158 (13)
C27	0.0690 (16)	0.0511 (14)	0.0384 (12)	−0.0059 (12)	0.0007 (11)	0.0003 (10)
C28	0.089 (2)	0.0719 (19)	0.0450 (14)	−0.0121 (16)	0.0092 (13)	0.0067 (13)
C29	0.096 (2)	0.070 (2)	0.0637 (18)	−0.0143 (17)	0.0111 (16)	0.0224 (15)
C30	0.082 (2)	0.0439 (14)	0.0726 (19)	−0.0097 (13)	0.0017 (15)	0.0126 (13)
C31	0.0491 (12)	0.0392 (11)	0.0492 (13)	−0.0016 (9)	−0.0065 (10)	0.0040 (9)
C32	0.0435 (12)	0.0444 (12)	0.0493 (12)	0.0125 (9)	−0.0037 (10)	−0.0103 (10)
C33	0.0394 (11)	0.0567 (14)	0.0409 (11)	0.0115 (10)	0.0003 (9)	−0.0100 (10)
C34	0.0340 (10)	0.0555 (13)	0.0360 (10)	0.0070 (9)	0.0019 (8)	−0.0025 (9)
C35	0.0566 (15)	0.0702 (17)	0.0660 (17)	0.0131 (13)	0.0206 (13)	0.0128 (14)
C36	0.0652 (19)	0.100 (3)	0.080 (2)	0.0059 (17)	0.0345 (16)	0.0147 (18)
C37	0.0507 (16)	0.111 (3)	0.074 (2)	0.0152 (17)	0.0236 (14)	−0.0060 (18)
C38	0.0495 (14)	0.0767 (19)	0.0624 (16)	0.0204 (13)	0.0094 (12)	−0.0118 (14)
C39	0.094 (2)	0.0494 (15)	0.078 (2)	0.0200 (15)	0.0102 (17)	−0.0190 (14)
C40	0.0425 (12)	0.0482 (12)	0.0436 (12)	0.0088 (10)	−0.0067 (9)	0.0004 (10)
C41	0.0378 (12)	0.0688 (17)	0.0548 (14)	0.0089 (11)	0.0007 (10)	0.0079 (12)
C42	0.0444 (13)	0.0656 (16)	0.0607 (15)	−0.0097 (12)	0.0030 (11)	0.0147 (13)
C43	0.0476 (13)	0.0449 (12)	0.0535 (13)	−0.0066 (10)	0.0030 (10)	0.0093 (10)
C44	0.0439 (11)	0.0400 (11)	0.0329 (10)	−0.0003 (9)	−0.0020 (8)	0.0040 (8)
C45	0.0479 (12)	0.0369 (11)	0.0498 (12)	0.0046 (9)	0.0062 (10)	−0.0108 (9)
C46	0.0439 (11)	0.0411 (11)	0.0398 (11)	0.0052 (9)	0.0089 (9)	−0.0037 (9)
C47	0.0393 (10)	0.0376 (10)	0.0320 (10)	0.0037 (8)	0.0056 (8)	0.0027 (8)
C48	0.0485 (13)	0.0405 (12)	0.0583 (14)	−0.0014 (10)	0.0130 (11)	0.0020 (10)
C49	0.0530 (14)	0.0489 (14)	0.086 (2)	0.0002 (11)	0.0315 (14)	0.0124 (13)
C50	0.0682 (17)	0.0594 (16)	0.0756 (18)	0.0093 (13)	0.0419 (15)	0.0048 (14)
C51	0.0643 (16)	0.0535 (14)	0.0561 (14)	0.0076 (12)	0.0223 (12)	−0.0108 (11)
C52	0.0608 (17)	0.0461 (15)	0.149 (3)	−0.0009 (13)	0.0253 (19)	−0.0377 (18)
O9A	0.171 (12)	0.067 (7)	0.154 (16)	−0.005 (7)	0.102 (11)	−0.022 (9)
O10A	0.267 (19)	0.142 (16)	0.045 (6)	−0.051 (13)	0.032 (8)	−0.018 (7)
O11A	0.205 (17)	0.050 (8)	0.179 (19)	−0.004 (9)	−0.062 (15)	0.052 (11)

### Geometric parameters (Å, º)

**Table:**

Ni1—O2	2.0371 (14)	C13—H13A	0.9600
Ni1—N4	2.0388 (16)	C13—H13B	0.9600
Ni1—O1	2.0483 (13)	C13—H13C	0.9600
Ni1—N1	2.0500 (16)	C14—C15	1.373 (3)
Ni1—N2	2.0501 (16)	C14—H14	0.9300
Ni1—N5	2.0564 (17)	C15—C16	1.382 (4)
Ni2—O4	2.0336 (14)	C15—H15	0.9300
Ni2—N10	2.0337 (17)	C16—C17	1.368 (4)
Ni2—N7	2.0455 (17)	C16—H16	0.9300
Ni2—N8	2.0594 (18)	C17—C18	1.399 (3)
Ni2—N11	2.0606 (17)	C17—H17	0.9300
Ni2—O3	2.0667 (14)	C19—C20	1.470 (3)
O1—C12	1.342 (2)	C19—C26	1.504 (3)
O1—H1O	0.8200	C20—C25	1.409 (3)
O2—C21	1.340 (2)	C20—C21	1.410 (3)
O3—C34	1.353 (2)	C21—C22	1.392 (4)
O3—H3O	0.8200	C22—C23	1.375 (4)
O4—C47	1.343 (2)	C22—H22	0.9300
O5W—H5WA	0.8355	C23—C24	1.361 (5)
O5W—H5WB	0.7365	C23—H23	0.9300
O6—N14	1.201 (4)	C24—C25	1.378 (5)
O7—N14	1.219 (3)	C24—H24	0.9300
O8—N14	1.215 (4)	C25—H25	0.9300
O9—N13	1.244 (4)	C26—H26A	0.9600
O10—N13	1.203 (3)	C26—H26B	0.9600
O11—N13	1.225 (4)	C26—H26C	0.9600
N1—C5	1.338 (3)	C27—C28	1.372 (4)
N1—C1	1.342 (3)	C27—H27	0.9300
N2—C6	1.284 (2)	C28—C29	1.385 (4)
N2—N3	1.385 (2)	C28—H28	0.9300
N3—C5	1.376 (3)	C29—C30	1.369 (4)
N3—H3N	0.8600	C29—H29	0.9300
N4—C19	1.296 (3)	C30—C31	1.396 (3)
N4—N6	1.371 (2)	C30—H30	0.9300
N5—C18	1.343 (3)	C32—C33	1.473 (4)
N5—C14	1.345 (3)	C32—C39	1.506 (3)
N6—C18	1.371 (3)	C33—C38	1.409 (3)
N6—H6N	0.8600	C33—C34	1.415 (3)
N7—C32	1.296 (3)	C34—C35	1.385 (4)
N7—N9	1.375 (3)	C35—C36	1.384 (4)
N8—C31	1.339 (3)	C35—H35	0.9300
N8—C27	1.348 (3)	C36—C37	1.368 (5)
N9—C31	1.374 (3)	C36—H36	0.9300
N9—H9N	0.8600	C37—C38	1.372 (5)
N10—C45	1.293 (3)	C37—H37	0.9300
N10—N12	1.376 (2)	C38—H38	0.9300
N11—C44	1.338 (3)	C39—H39A	0.9600
N11—C40	1.345 (3)	C39—H39B	0.9600
N12—C44	1.365 (3)	C39—H39C	0.9600
N12—H12N	0.8600	C40—C41	1.369 (3)
N13—O11A	1.127 (12)	C40—H40	0.9300
N13—O9A	1.169 (11)	C41—C42	1.387 (4)
N13—O10A	1.278 (10)	C41—H41	0.9300
C1—C2	1.366 (3)	C42—C43	1.362 (4)
C1—H1	0.9300	C42—H42	0.9300
C2—C3	1.378 (4)	C43—C44	1.398 (3)
C2—H2	0.9300	C43—H43	0.9300
C3—C4	1.364 (4)	C45—C46	1.470 (3)
C3—H3	0.9300	C45—C52	1.501 (3)
C4—C5	1.395 (3)	C46—C51	1.406 (3)
C4—H4	0.9300	C46—C47	1.419 (3)
C6—C7	1.480 (3)	C47—C48	1.400 (3)
C6—C13	1.501 (3)	C48—C49	1.378 (3)
C7—C8	1.403 (3)	C48—H48	0.9300
C7—C12	1.408 (3)	C49—C50	1.382 (4)
C8—C9	1.377 (3)	C49—H49	0.9300
C8—H8	0.9300	C50—C51	1.376 (4)
C9—C10	1.372 (4)	C50—H50	0.9300
C9—H9	0.9300	C51—H51	0.9300
C10—C11	1.382 (3)	C52—H52A	0.9600
C10—H10	0.9300	C52—H52B	0.9600
C11—C12	1.396 (3)	C52—H52C	0.9600
C11—H11	0.9300		
			
O2—Ni1—N4	86.35 (6)	N5—C14—C15	123.3 (2)
O2—Ni1—O1	84.90 (5)	N5—C14—H14	118.3
N4—Ni1—O1	98.37 (6)	C15—C14—H14	118.3
O2—Ni1—N1	91.74 (6)	C14—C15—C16	117.9 (2)
N4—Ni1—N1	95.58 (7)	C14—C15—H15	121.0
O1—Ni1—N1	165.40 (6)	C16—C15—H15	121.0
O2—Ni1—N2	90.53 (6)	C17—C16—C15	120.5 (2)
N4—Ni1—N2	173.93 (6)	C17—C16—H16	119.8
O1—Ni1—N2	86.53 (6)	C15—C16—H16	119.8
N1—Ni1—N2	79.29 (6)	C16—C17—C18	118.1 (2)
O2—Ni1—N5	160.88 (6)	C16—C17—H17	120.9
N4—Ni1—N5	79.64 (7)	C18—C17—H17	120.9
O1—Ni1—N5	84.34 (6)	N5—C18—N6	116.85 (18)
N1—Ni1—N5	102.46 (7)	N5—C18—C17	122.3 (2)
N2—Ni1—N5	104.53 (6)	N6—C18—C17	120.89 (19)
O4—Ni2—N10	85.87 (6)	N4—C19—C20	119.27 (19)
O4—Ni2—N7	97.46 (6)	N4—C19—C26	120.6 (2)
N10—Ni2—N7	176.60 (7)	C20—C19—C26	120.1 (2)
O4—Ni2—N8	87.37 (7)	C25—C20—C21	117.7 (2)
N10—Ni2—N8	101.56 (7)	C25—C20—C19	117.7 (2)
N7—Ni2—N8	79.35 (7)	C21—C20—C19	124.59 (19)
O4—Ni2—N11	163.23 (6)	O2—C21—C22	118.8 (2)
N10—Ni2—N11	79.71 (7)	O2—C21—C20	122.0 (2)
N7—Ni2—N11	96.89 (7)	C22—C21—C20	119.1 (2)
N8—Ni2—N11	103.78 (7)	C23—C22—C21	121.5 (3)
O4—Ni2—O3	84.32 (6)	C23—C22—H22	119.3
N10—Ni2—O3	95.00 (6)	C21—C22—H22	119.3
N7—Ni2—O3	84.68 (7)	C24—C23—C22	120.2 (3)
N8—Ni2—O3	160.88 (7)	C24—C23—H23	119.9
N11—Ni2—O3	88.43 (6)	C22—C23—H23	119.9
C12—O1—Ni1	122.46 (11)	C23—C24—C25	120.0 (3)
C12—O1—H1O	109.5	C23—C24—H24	120.0
Ni1—O1—H1O	128.1	C25—C24—H24	120.0
C21—O2—Ni1	119.64 (13)	C24—C25—C20	121.6 (3)
C34—O3—Ni2	119.85 (12)	C24—C25—H25	119.2
C34—O3—H3O	109.5	C20—C25—H25	119.2
Ni2—O3—H3O	130.6	C19—C26—H26A	109.5
C47—O4—Ni2	123.72 (12)	C19—C26—H26B	109.5
H5WA—O5W—H5WB	112.2	H26A—C26—H26B	109.5
C5—N1—C1	118.75 (18)	C19—C26—H26C	109.5
C5—N1—Ni1	112.14 (13)	H26A—C26—H26C	109.5
C1—N1—Ni1	128.78 (15)	H26B—C26—H26C	109.5
C6—N2—N3	119.17 (16)	N8—C27—C28	123.1 (3)
C6—N2—Ni1	128.72 (14)	N8—C27—H27	118.5
N3—N2—Ni1	109.51 (11)	C28—C27—H27	118.5
C5—N3—N2	116.15 (16)	C27—C28—C29	118.0 (3)
C5—N3—H3N	121.9	C27—C28—H28	121.0
N2—N3—H3N	121.9	C29—C28—H28	121.0
C19—N4—N6	119.74 (17)	C30—C29—C28	120.3 (3)
C19—N4—Ni1	128.82 (15)	C30—C29—H29	119.9
N6—N4—Ni1	111.42 (12)	C28—C29—H29	119.9
C18—N5—C14	117.87 (18)	C29—C30—C31	118.3 (3)
C18—N5—Ni1	112.01 (13)	C29—C30—H30	120.9
C14—N5—Ni1	127.52 (14)	C31—C30—H30	120.9
C18—N6—N4	117.93 (16)	N8—C31—N9	116.8 (2)
C18—N6—H6N	121.0	N8—C31—C30	122.2 (2)
N4—N6—H6N	121.0	N9—C31—C30	121.0 (2)
C32—N7—N9	119.99 (19)	N7—C32—C33	119.4 (2)
C32—N7—Ni2	129.32 (16)	N7—C32—C39	120.7 (2)
N9—N7—Ni2	110.66 (13)	C33—C32—C39	119.8 (2)
C31—N8—C27	118.2 (2)	C38—C33—C34	116.8 (2)
C31—N8—Ni2	112.50 (15)	C38—C33—C32	118.6 (2)
C27—N8—Ni2	128.24 (16)	C34—C33—C32	124.5 (2)
C31—N9—N7	117.86 (18)	O3—C34—C35	119.9 (2)
C31—N9—H9N	121.1	O3—C34—C33	120.4 (2)
N7—N9—H9N	121.1	C35—C34—C33	119.7 (2)
C45—N10—N12	119.45 (18)	C36—C35—C34	121.3 (3)
C45—N10—Ni2	130.17 (15)	C36—C35—H35	119.3
N12—N10—Ni2	110.10 (12)	C34—C35—H35	119.3
C44—N11—C40	118.15 (19)	C37—C36—C35	119.9 (3)
C44—N11—Ni2	111.44 (14)	C37—C36—H36	120.0
C40—N11—Ni2	129.31 (15)	C35—C36—H36	120.0
C44—N12—N10	117.91 (17)	C36—C37—C38	119.7 (3)
C44—N12—H12N	121.0	C36—C37—H37	120.2
N10—N12—H12N	121.0	C38—C37—H37	120.2
O11A—N13—O9A	132.6 (15)	C37—C38—C33	122.5 (3)
O10—N13—O11	126.1 (4)	C37—C38—H38	118.7
O10—N13—O9	117.4 (3)	C33—C38—H38	118.7
O11—N13—O9	116.3 (3)	C32—C39—H39A	109.5
O11A—N13—O10A	113.8 (14)	C32—C39—H39B	109.5
O9A—N13—O10A	112.7 (12)	H39A—C39—H39B	109.5
O6—N14—O8	117.6 (3)	C32—C39—H39C	109.5
O6—N14—O7	119.6 (4)	H39A—C39—H39C	109.5
O8—N14—O7	122.3 (4)	H39B—C39—H39C	109.5
N1—C1—C2	122.9 (2)	N11—C40—C41	122.9 (2)
N1—C1—H1	118.6	N11—C40—H40	118.6
C2—C1—H1	118.6	C41—C40—H40	118.6
C1—C2—C3	117.8 (2)	C40—C41—C42	118.4 (2)
C1—C2—H2	121.1	C40—C41—H41	120.8
C3—C2—H2	121.1	C42—C41—H41	120.8
C4—C3—C2	120.9 (2)	C43—C42—C41	119.9 (2)
C4—C3—H3	119.5	C43—C42—H42	120.1
C2—C3—H3	119.5	C41—C42—H42	120.1
C3—C4—C5	118.0 (2)	C42—C43—C44	118.4 (2)
C3—C4—H4	121.0	C42—C43—H43	120.8
C5—C4—H4	121.0	C44—C43—H43	120.8
N1—C5—N3	117.42 (17)	N11—C44—N12	117.27 (19)
N1—C5—C4	121.7 (2)	N11—C44—C43	122.2 (2)
N3—C5—C4	120.9 (2)	N12—C44—C43	120.5 (2)
N2—C6—C7	118.40 (17)	N10—C45—C46	119.86 (19)
N2—C6—C13	122.74 (19)	N10—C45—C52	120.8 (2)
C7—C6—C13	118.85 (18)	C46—C45—C52	119.3 (2)
C8—C7—C12	118.13 (19)	C51—C46—C47	117.5 (2)
C8—C7—C6	117.94 (18)	C51—C46—C45	118.7 (2)
C12—C7—C6	123.91 (17)	C47—C46—C45	123.84 (19)
C9—C8—C7	121.8 (2)	O4—C47—C48	119.26 (19)
C9—C8—H8	119.1	O4—C47—C46	121.81 (19)
C7—C8—H8	119.1	C48—C47—C46	118.91 (19)
C10—C9—C8	119.5 (2)	C49—C48—C47	121.5 (2)
C10—C9—H9	120.3	C49—C48—H48	119.2
C8—C9—H9	120.3	C47—C48—H48	119.2
C9—C10—C11	120.4 (2)	C48—C49—C50	120.1 (2)
C9—C10—H10	119.8	C48—C49—H49	120.0
C11—C10—H10	119.8	C50—C49—H49	120.0
C10—C11—C12	120.9 (2)	C51—C50—C49	119.3 (2)
C10—C11—H11	119.6	C51—C50—H50	120.3
C12—C11—H11	119.6	C49—C50—H50	120.3
O1—C12—C11	119.95 (18)	C50—C51—C46	122.4 (2)
O1—C12—C7	120.92 (17)	C50—C51—H51	118.8
C11—C12—C7	119.14 (18)	C46—C51—H51	118.8
C6—C13—H13A	109.5	C45—C52—H52A	109.5
C6—C13—H13B	109.5	C45—C52—H52B	109.5
H13A—C13—H13B	109.5	H52A—C52—H52B	109.5
C6—C13—H13C	109.5	C45—C52—H52C	109.5
H13A—C13—H13C	109.5	H52A—C52—H52C	109.5
H13B—C13—H13C	109.5	H52B—C52—H52C	109.5

### Hydrogen-bond geometry (Å, º)

**Table:**

*D*—H···*A*	*D*—H	H···*A*	*D*···*A*	*D*—H···*A*
O1—H1*O*···O4	0.82	1.62	2.4093 (18)	161
O3—H3*O*···O2	0.82	1.66	2.4647 (19)	167
O5*W*—H5*WA*···O11^i^	0.84	2.16	2.979 (5)	165
O5*W*—H5*WA*···O11*A*^i^	0.84	1.94	2.767 (14)	170
O5*W*—H5*WB*···O6^i^	0.74	2.47	3.142 (4)	153
O5*W*—H5*WB*···O7^i^	0.74	2.42	3.094 (5)	153
N3—H3*N*···O5*W*	0.86	2.23	2.933 (2)	139
N6—H6*N*···O11	0.86	2.19	2.991 (4)	156
N6—H6*N*···O11*A*	0.86	2.62	3.47 (3)	172
N9—H9*N*···O10^ii^	0.86	2.30	3.041 (4)	145
N9—H9*N*···O9*A*^ii^	0.86	2.26	3.107 (12)	167
N12—H12*N*···O6^iii^	0.86	2.13	2.961 (3)	162
C2—H2···O10^iv^	0.93	2.63	3.437 (5)	146
C4—H4···O6^i^	0.93	2.56	3.409 (4)	152
C13—H13*C*···O9^i^	0.96	2.33	3.231 (5)	156
C15—H15···O8^v^	0.93	2.62	3.544 (4)	170
C26—H26*C*···O10*A*	0.96	2.59	3.332 (18)	134
C28—H28···O10*A*^vi^	0.93	2.64	3.104 (12)	111
C30—H30···O10^ii^	0.93	2.33	3.117 (5)	142
C39—H39*A*···O9*A*^ii^	0.96	2.39	2.938 (11)	116

Symmetry codes: (i) *x*−1/2, −*y*+1/2, *z*+1/2; (ii) −*x*+3/2, *y*+1/2, −*z*+1/2; (iii) −*x*+3/2, *y*−1/2, −*z*+1/2; (iv) −*x*+2, −*y*, −*z*+1; (v) −*x*+2, −*y*+1, −*z*+1; (vi) *x*−1/2, −*y*+1/2, *z*−1/2.
